# Histamine, Neuroinflammation and Neurodevelopment: A Review

**DOI:** 10.3389/fnins.2021.680214

**Published:** 2021-07-14

**Authors:** Elliott Carthy, Tommas Ellender

**Affiliations:** ^1^Department of Pharmacology, University of Oxford, Oxford, United Kingdom; ^2^Department of Biomedical Sciences, University of Antwerp, Antwerp, Belgium

**Keywords:** histamine, brain, mast cells, microglia, neurodevelopment, neuroinflammation, astrocytes, neurodevelopmental disorders

## Abstract

The biogenic amine, histamine, has been shown to critically modulate inflammatory processes as well as the properties of neurons and synapses in the brain, and is also implicated in the emergence of neurodevelopmental disorders. Indeed, a reduction in the synthesis of this neuromodulator has been associated with the disorders Tourette’s syndrome and obsessive-compulsive disorder, with evidence that this may be through the disruption of the corticostriatal circuitry during development. Furthermore, neuroinflammation has been associated with alterations in brain development, e.g., impacting synaptic plasticity and synaptogenesis, and there are suggestions that histamine deficiency may leave the developing brain more vulnerable to proinflammatory insults. While most studies have focused on neuronal sources of histamine it remains unclear to what extent other (non-neuronal) sources of histamine, e.g., from mast cells and other sources, can impact brain development. The few studies that have started exploring this *in vitro*, and more limited *in vivo*, would indicate that non-neuronal released histamine and other preformed mediators can influence microglial-mediated neuroinflammation which can impact brain development. In this Review we will summarize the state of the field with regard to non-neuronal sources of histamine and its impact on both neuroinflammation and brain development in key neural circuits that underpin neurodevelopmental disorders. We will also discuss whether histamine receptor modulators have been efficacious in the treatment of neurodevelopmental disorders in both preclinical and clinical studies. This could represent an important area of future research as early modulation of histamine from neuronal as well as non-neuronal sources may provide novel therapeutic targets in these disorders.

## Introduction

Histamine is a biogenic monoamine as well as an endogenous neurotransmitter with a diverse array of physiological functions, ranging from local inflammatory responses to regulating synaptic transmission in the brain. A select number of mammalian cell types synthesize histamine, including those of the immune system (Schwartz, 1987; Dvorak, 1997), such as mast cells (and basophils, platelets), as well as small populations of neurons in the brain (Haas and Panula, 2003), such a those found in the tuberomammillary nucleus (TMN) of the hypothalamus. In the human adult brain, there are approximately 64,000 histaminergic neurons located in and around the TMN (Haas et al., 2008). These project diffusely throughout all areas of the brain, differentially modulating many distinct neuronal circuits (Blandina et al., 2012). Once released histamine can have widespread effects throughout the brain at large numbers of different cell types that express one or more of the four known histamine G protein-coupled receptors (Panula et al., 2015).

**FIGURE 1:**
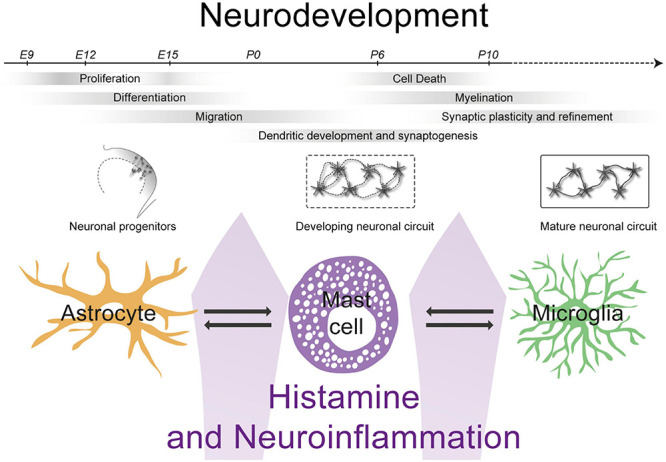
The interactions between mast cells, astrocytes and microglia can impact levels of histamine and neuroinflammation influencing neurodevelopment.

A wealth of data has demonstrated clear roles for histamine acting directly on neurons and neuronal structures (Haas and Panula, 2003; Haas et al., 2008; Panula and Nuutinen, 2013; Shan et al., 2015; Bolam and Ellender, 2016; Yoshikawa et al., 2021), but much less is known how histamine can act on other cell types found in the brain. Indeed, the young developing brain exhibits high numbers of some of these other cell types (e.g., microglia and mast cells) whose numbers change dynamically during development. Moreover, the young developing brain also contains a permeable blood brain barrier resulting in the potential for access of systemic factors and immune cells to act on developing neurons and synapses. In this Review, we will focus on three different non-neuronal cell types found in the brain, the microglia, mast cells and astrocytes and their interaction with histamine. We will discuss how they are involved in histaminergic signaling in the brain and how aberrant behavior of these cell types can impact on the development of neurons and synapses (see Figure 1). This is of importance as histamine has been implicated in a variety of early onset neurodevelopmental disorders. Furthermore, we will also discuss whether histamine receptor modulators have been efficacious in the treatment of difficulties associated with neurodevelopmental disorders in both preclinical and clinical studies.

## Histamine and the Developing Brain

### Histamine Synthesis and Metabolism

Histamine is synthesized from the amino acid, histidine. Histidine is one of the nine essential amino acids humans must get from their diet and is present in most protein-rich foods (e.g., fish, eggs, and nuts). Histidine undergoes oxidative decarboxylation by the enzyme histidine decarboxylase (*Hdc*), which is expressed by a select number of cells in the brain, including the histaminergic neurons in the TMN of the hypothalamus (Haas and Panula, 2003), transiently by other young developing neurons (Nissinen et al., 1995; Karlstedt et al., 2001a), but also by many non-neuronal cells including microglia and mast cells (Auvinen and Panula, 1988). Unlike for the other monoamines, no high-affinity histamine reuptake system has yet been identified in the mammalian brain [but see for Drosophila (Borycz et al., 2002; Chaturvedi et al., 2014; Stenesen et al., 2015; Xu et al., 2015)]. Although astrocytic plasma membrane monoamine and low-affinity organic cation transporters play a role in the uptake of histamine (Amphoux et al., 2006; Yoshikawa et al., 2013), this suggests that histamine is likely degraded in the extracellular space (Schwartz et al., 1991; Haas and Panula, 2003) with a possible half-life ranging in minutes (Schwartz et al., 1991). Inactivation of histamine within the central nervous system (CNS) occurs via methylation to inactive tele-methylhistamine by the enzyme histamine *N*-methyltransferase (HNMT) (Bowsher et al., 1983; Barnes and Hough, 2002; Haas et al., 2008) [see for recent review (Yoshikawa et al., 2021)]. Once released, histamine has multiple pleiotropic functions within the developing and mature central nervous system, including regulating neuronal excitability and neurotransmission (Haas et al., 2008), synaptic plasticity (Han et al., 2020), and immunomodulation (Forsythe, 2019) and has a functional role in modulating many aspects of behavior including learning, cognition, wakefulness, attention and memory (Haas and Panula, 2003; Haas et al., 2008; Passani et al., 2014). Given its effect at promoting wakefulness and attention, it will come as no surprise that histaminergic neuronal firing rates are highest during waking hours and low or silent during sleep (Takahashi et al., 2006; Lin et al., 2011) and that increases in histaminergic firing have been observed during periods of increased attention (Takahashi et al., 2006) and motivated behaviors (Torrealba et al., 2012).

### Sources of Histamine in the Developing Brain

Histamine has been shown to be one of the earliest neuromodulators present within the developing embryonic rodent brain, with a prenatal peak seen around embryonic day (E)14 – 16 (Vanhala et al., 1994). Histamine-immunoreactive neurons can be found within various parts of the developing brain, including the rhombencephalon, mesencephalon, and in some regions of the diencephalon (Auvinen and Panula, 1988). These are a transient source of histamine, the peak of which coincides with periods of increased neurogenesis and the initial phase of gliogenesis, suggesting it may modulate these early processes (Sauvageot and Stiles, 2002; Molina-Hernández and Velasco, 2008; Rodríguez-Martínez et al., 2012). In addition, transient sources of histamine in the brain include a subgroup of serotonergic neurons from the raphe nucleus that project widely in the brain and transiently co-express the synthesizing enzyme *Hdc* as well as histamine during early embryonic development (Wada et al., 1991; Vanhala et al., 1994; Molina-Hernández et al., 2012), and a population of thalamic neurons that also transiently expresses *Hdc* (Nissinen et al., 1995; Nissinen and Panula, 1995; Karlstedt et al., 2001a; Zecharia et al., 2012). The number of histamine-immunoreactive neurons in these regions then steadily decreases until E18, after which the histaminergic neurons of the TMN of the hypothalamus become detectable at E20 which project widely throughout the brain and form a main neuronal histaminergic source that persist into adulthood (Auvinen and Panula, 1988; Haas et al., 2008; Molina-Hernández et al., 2012). Neuronal histamine is stored within both the cell somata as well as within vesicles in axonal varicosities as packaged by the vesicular monoamine transporter (VMAT2) and is released by activated neurons (Haas et al., 2008; Puttonen et al., 2017). In addition, ependymal cells lining the ventricles also likely synthesize histamine as they express the synthesizing enzyme *Hdc* (Karlstedt et al., 2001a). Lastly, immune cells, including mast cells and microglia encompass non-neuronal sources of histamine (Katoh et al., 2001; Haas et al., 2008), which store histamine in granules, which are released when appropriately activated (Forsythe, 2019). We’ll mainly focus on these other non-neuronal sources of histamine in this Review.

### Histamine Receptors

Histamine acts at both neuronal and non-neuronal cells in the brain at one or more of the four known histamine G protein-coupled receptors denoted as the H_1_–H_4_ receptors (Passani and Blandina, 2011; Panula et al., 2015; Haas and Panula, 2016). Three of the four histamine receptors (H_1_, H_2_, and H_3_ receptors) are widely expressed throughout the CNS (Haas et al., 2008; Molina-Hernández et al., 2012; Panula and Nuutinen, 2013; Haas and Panula, 2016). The H_1_ receptor is coupled to an intracellular G_*q*_ protein that activates phospholipase C and inositol triphosphate (IP3) signaling pathways. The H_1_ receptor has been identified in the developing rat CNS from E14 within the telencephalon, mesencephalon and the spinal cord (Kinnunen et al., 1998) and expression is retained into adulthood. The H_2_ receptors are coupled to G_*s*_ second messenger proteins that increase the production of cyclic adenosine monophosphate (cAMP) and subsequent activation of protein kinase A. The H_2_ receptor is expressed early by neural stem cells at E12 and by neurons within the raphe nuclei, with subsequent uniform expression found throughout the rat brain by E15 (Molina-Hernández et al., 2012) with prominent expression in the cortical plate by E17 (Karlstedt et al., 2001b). The H_3_ receptors are coupled to G_*i*_ second messenger proteins that have high constitutive activity and their expression is initially more restricted within the developing brain (Karlstedt et al., 2003; Molina-Hernández et al., 2012). At E15, they are predominantly expressed within the midbrain, medulla and spinal cord, with expression in the latter two diminishing after E16 and expression within the hypothalamus and nucleus accumbens subsequently increasing. By E19, H_3_ receptors are expressed within the cortical plate and deep cortical layers. H_3_ receptors have been shown to control the biosynthesis (Arrang et al., 1983; Arrang et al., 1987; Haas et al., 2008) and release of histamine as autoreceptors (Torrent et al., 2005; Arrang et al., 2007; Haas et al., 2008) and also function as hetero-receptors to regulate the release of other neurotransmitters including acetylcholine, glutamate, GABA, serotonin, and dopamine (Molina-Hernández et al., 2012). The H_4_ receptor is also coupled to intracellular G_*i*_ protein signaling (Gbahou et al., 2006) but little is known about H_4_ receptor expression in the developing brain and if and what role it may have in influencing neurodevelopment. There have been inconsistent findings regarding the expression of H_4_ receptors in the brain (Schneider and Seifert, 2016). Recent qRT-PCR experiments of striatal tissue of mice at different developmental periods (postnatal day 3 onward) showed no evidence for H_4_ receptor expression whereas H_1_, H_2_, and H_3_ receptor were detected from the first postnatal week onward (Han et al., 2020). However, some studies using other methods have detected expression in the amygdala, hippocampus, striatum, substantial nigra, thalamus, and hypothalamus (Connelly et al., 2009; Strakhova et al., 2009) and on some cell lines of microglia (Ferreira et al., 2012) and mast cells (Thangam et al., 2018).

### Histamine and Developing Neurons and Synapses

Histamine has many different actions on the nervous system and has been shown to act at neuronal cells from the earliest developmental periods onward (see Table 1) (Molina-Hernández et al., 2012; Panula and Nuutinen, 2013; Panula et al., 2014). For example, histamine can increase the proliferation of rodent neural stem cells (NSC) or progenitors expressing histamine receptors during both embryonic and postnatal periods and increase the differentiation of *adult* NSCs into GABAergic neurons and the differentiation of *fetal* NSCs into glutamatergic FOXP2-positive neurons (Molina-Hernández and Velasco, 2008; Bernardino et al., 2012; Rodríguez-Martínez et al., 2012; Eiriz et al., 2014). Subsequently histamine also impacts the number and length of ramifications of neurites of developing neurons (Bernardino et al., 2012), suggesting it can modulate neuronal dendritic and axonal maturation. Recent studies have highlighted further roles for histamine during later periods of brain development impacting the formation of neuronal circuits in both the basal ganglia (Han et al., 2020) as well as the cortex (*Prof. Molina-Hernandez - personal communication*). The basal ganglia are a collection of subcortical nuclei that have crucial roles for motor behavior and cognition (Graybiel et al., 1994; Grillner et al., 2005). The striatum is the major input nucleus of the basal ganglia which both during early postnatal periods (Han et al., 2020) and in adulthood expresses high numbers of H_1_, H_2_, and H_3_ receptors (Haas and Panula, 2003), suggesting that histamine can act as a modulator of many processes in the basal ganglia (Bolam and Ellender, 2016). In both the striatum, and more generally in the brain, the first postnatal weeks are characterized by rapid increases in the number and strength of excitatory synaptic connections (Tepper et al., 1998; Kozorovitskiy et al., 2012; Peixoto et al., 2016; Krajeski et al., 2019; Peixoto et al., 2019) and these connections exhibit plastic changes (i.e., synaptic plasticity) which is crucial for their development and refinement (Calabresi et al., 1992a; Kreitzer and Malenka, 2008). Han et al. (2020) found that histamine could facilitate NMDA-receptor dependent synaptic plasticity at corticostriatal synapses during the second postnatal week, which was dependent on the H_3_ receptor, potentially through histamine-mediated dendritic depolarization (Calabresi et al., 1992b). Interestingly, histamine inhibited the NMDA-receptor dependent synaptic plasticity at older ages, again through the H_3_ receptor, which could indicate differential coupling of the H_3_ receptor to intracellular pathways depending on age (Drutel et al., 2001; Rapanelli et al., 2016). This suggests that histamine is key in gating corticostriatal synaptic plasticity at critical periods of remodeling and it remains to be investigated whether it might play a similar role in other developing neuronal circuits also. As discussed in greater detail in these Reviews (Molina-Hernández et al., 2012; Panula and Nuutinen, 2013; Panula et al., 2014) the main impacts of histamine on neurons and synapses is the modulation of their excitability, often through action at H_1_ and/or H_2_ receptors (Munakata and Akaike, 1994; Prast et al., 1999; Ellender et al., 2011), as well as the modulation of release of various neurotransmitters through action at H_3_ receptors (Brown and Haas, 1999; Doreulee et al., 2001; Molina-Hernández et al., 2001; Ellender et al., 2011). Most of these studies are performed in rodents during the first months of postnatal life, which corresponds to the second and third gestational trimester in humans (Romijn et al., 1991; Clancy et al., 2001). Indeed, as rodents are born at very immature stages of brain development their brains undergo most maturation postnatally suggesting findings have relevance for our understanding of human brain development.

**TABLE 1:** Effect of histamine on neurons, microglia, astrocytes and mast cells during early brain development.

	**Effect of histamine**	**References**
**Neurons**	Increased neural stem cell differentiation into GABAergic (*adult*) or glutamatergic (*fetal*) neurons via H_1_ receptors.	Molina-Hernández and Velasco, 2008; Bernardino et al., 2012
	Neural stem cell proliferation, apoptosis and decreased glial cell differentiation within the neuroepithelium of the cortex via H_2_ receptors.	Molina-Hernández and Velasco, 2008; Bernardino et al., 2012; Rodríguez-Martínez et al., 2012
	Modulation of synaptic plasticity and neural circuits.	
	Increased ACh release from striatal cholinergic interneurons via H_1_ or H_2_ receptors.	Prast et al., 1999
	Inhibition of GABA release from spiny projection neurons in the striatum via H_3_ receptors.	Ellender et al., 2011
	Inhibition of glutamate release from synaptosomes.	Molina-Hernández et al., 2001
	Inhibition of glutamate release from cortical afferents to striatum.	Doreulee et al., 2001; Ellender et al., 2011
	Facilitating synaptic plasticity at corticostriatal synapses from the second postnatal week onward via H_3_ receptors.	Han et al., 2020
	Facilitating long-term potentiation in the CA1 of the hippocampus via H_1_ and H_2_ receptors.	Brown et al., 1995; Dai et al., 2007; Haas et al., 2008; Chepkova et al., 2012
**Microglia**	Microglia express all four subsets of histamine receptor.	Dong et al., 2014a; Haas and Panula, 2016; Zhang et al., 2020
	Histamine can induce migration of microglia though H_4_ receptor activation.	Ferreira et al., 2012; Dong et al., 2014a; Fang et al., 2020; Zhang et al., 2020
	Histamine can induce both pro- and anti-inflammatory response from microglia via H_1_ and H_4_ receptors.	Rocha et al., 2016; Lenz et al., 2018
	Promote phagocytosis via H_1_ receptor activation and the production of reactive oxygen species and prostaglandin E2.	Frick et al., 2016
	*Hdc* knockout (KO) mice have a normal number of microglia but with reduced ramifications, reduced IGF-1 expression and reduced expression of H_4_ receptor.	Frick et al., 2016
	Pro-inflammatory microglial response to challenge with LPS was greater in *Hdc* KO mice.	Iida et al., 2015
	H_3_ receptor mediated autocrine and paracrine signaling has also been shown to inhibit microglial chemotaxis and phagocytosis along with inhibiting LPS-induced cytokine production.	
**Astrocytes**	The H_1_, H_2_, and H_3_ receptors have been consistently shown to be expressed on astrocytes.	Jurič et al., 2016; Karpati et al., 2018
	H_3_ receptor expression may be restricted to certain brain regions and may vary depending on the species that is studied.	
	Elicit glutamate release in an H_1_ receptor-dependent and concentration-dependent manner.	Hosli and Hosli, 1984; Hosli et al., 1984
	Astrocytic Ca^2+^ signaling and subsequent astrocytic glutamate release was highly sensitive to histamine and concentration-dependent acting through the H_1_ receptor. Astrocytic cAMP levels increased in response to histamine, it remains unclear if and what role this has in gliotransmitter release.	Karpati et al., 2018
	Histamine can act synergistically with pro-inflammatory cytokines such as IL-1 and IL-6 to modulate astrocytic release of neurotrophins such as NGF.	Lipnik-Štangelj and Čarman-Kržan, 2005; Lipnik-Stangelj and Carman-Krzan, 2006; Ales et al., 2008
	Histamine selectively upregulates the expression of H_1_, H_2_, and H_3_ receptors, stimulated the synthesis of astrocytic GDNF and concentration-dependent inhibition of the production of pro-inflammatory cytokines, TNF-α and IL-1β.	Xu et al., 2018
**Mast cells**	Mast cells are a non-neuronal source of histamine that can be released upon degranulation.	Katoh et al., 2001; Haas et al., 2008
	Mast cell expression of H_1_ and H_4_ receptors is implicated in type 1 hypersensitivity reactions. However, their expression in brain mast cells is not confirmed.	Thangam et al., 2018
	Co-cultures of mast cells with astrocytes has been shown to lead to the release of histamine and leukotrienes via CD40-CD40L interactions.	Kim et al., 2010
	The mast cell degranulator, C48/80 can trigger hypothalamic microglial activation and the release of IL-6 and TNF-α.	Dong et al., 2017
	Mast cell activation with estradiol stimulates microglial activation and subsequent prostaglandin release and that was associated with increased dendritic spine density and higher amounts of the dendritic spine protein, spinophilin.	Lenz et al., 2018
	Mast cells strongly adhere to hippocampal neurons via cell adhesion molecule 1d.	Hagiyama et al., 2011
	Neuropeptides released from neurites bind directly bind to mast cells, altering their activation state.	Kulka et al., 2008
	Mast cell derived products may enter neurons via transgranulation, whereby mast cells are in direct contact with neurons and exocytosed mast cell granules are directly taken up by the adjacent neuron.	Wilhelm et al., 2005

*ACh, acetylcholine; cAMP, cyclic adenosine monophosphate; GABA, gamma amino butyric acid; GDNF, glial-derived neurotrophic factor; H_1_, histamine 1; H_2_, histamine 2; H_3_, histamine 3; H_4_, histamine 4; Hdc KO, histamine decarboxylase knock out; IL-1β, interleukin-1 beta; IL-6, interleukin-6; LPS, lipopolysaccharide; NGF, nerve growth factor; NSC, neural stem cells; TNF-α, tumor necrosis factor alpha.*

Overall, histamine is an active neuromodulator in the developing brain acting at many developing cells and structures suggesting it may have an important role in brain development. Interestingly, although many of these cell express histamine receptors during early development, often the neuronal histaminergic afferents (e.g., from the TMN) might not be present yet (Auvinen and Panula, 1988; Han et al., 2020), suggesting that other histaminergic sources could be important during specific periods of development. As we will further outline below histaminergic dysregulation has been implicated in several neurodevelopmental (Ercan-Sencicek et al., 2010; Stevenson et al., 2010; Fernandez et al., 2012; Karagiannidis et al., 2013; Baldan et al., 2014; Wright et al., 2017) and a range of neuropsychiatric disorders (Shan et al., 2015).

## Histamine and Neurodevelopmental Disorders

Neurodevelopmental disorders (NDDs) comprise a heterogeneous group of highly heritable medical conditions that principally affect social communication, language, attention, impulsivity, learning, perception and motor coordination. They can frequently co-exist, persisting from childhood to adulthood with adverse medical and psychosocial outcomes on both the individual and their family (Jalenques et al., 2017). The clinical features of NDDs are thought to result, at least in part, from aberrant formation of key neuronal circuits during early periods of neurodevelopment, which can have lifelong effects. A growing body of literature implicates histamine dysregulation in NDDs including Tourette’s syndrome (TS), autism spectrum disorders (ASD), attention deficit hyperactivity disorder (ADHD), and schizophrenia and is highlighted in further detail below (see also Table 2).

**TABLE 2:** The role of histamine in the aetiology of neurodevelopmental disorders.

	**Role of histamine**	**References**
**Tourette’s syndrome**	A rare nonsense mutation in the gene encoding *Histidine decarboxylase (Hdc)*, the rate-limiting enzyme in histamine synthesis, has been implicated in a two-generation pedigree	Ercan-Sencicek et al., 2010
	There is a gene-dose dependent decrease in histamine concentration in the hypothalamus, striatum and cortex, in mice that were heterozygous or knock out for *Hdc* compared to wild type mice.	Baldan et al., 2014
	*Hdc* KO mice exhibit repetitive movements in response to acute stress and psychostimulant challenge that were mitigated with pre-treatment with the dopamine 2 receptor antagonist, haloperidol.	Baldan et al., 2014
	Dopamine levels and D_2_/D_3_ receptor expression are increased in *Hdc* KO mice, suggesting that there may be an interaction between histaminergic and dopaminergic signaling in mediating the symptoms of Tourette syndrome.	Baldan et al., 2014
	H_3_ receptors are upregulated in *Hdc* KO mice, and chemogenetic activation of these receptors in the dorsal striatum can precipitate stereotypies.	Rapanelli et al., 2017
	Histamine infusion may reduce the concentration of dopamine in the striatum by agonizing H_3_ heteroreceptors on dopaminergic afferents.	Schlicker et al., 1993
**Autism spectrum disorders**	Histamine dysregulation may have a role in mediating autism spectrum disorder phenotype with altered expression of key histamine signaling genes *HNMT*, *HRH1*, *HRH2*, and *HRH3* in post-mortem brains of patients with ASD.	Wright et al., 2017
	H_3_ receptor has been implicated in repetitive behavior-like pathology including stereotypies that may be a feature of ASDs.	Rapanelli et al., 2017
**Attention deficit hyperactivity disorder**	Methylphenidate, and atomoxetine may stimulate cortical histamine through enhanced dopamine and noradrenaline transmission.	Horner et al., 2007
	Polymorphisms of the *HNMT* gene have been seen in those with ADHD.	Stevenson et al., 2010
**Schizophrenia**	Elevated levels of the histamine metabolite tele-methylhistamine have been implicated in schizophrenia, suggesting greater histamine release and turnover.	Prell et al., 1995, 1996
	Adjunct use of the H_2_ receptor antagonist, ranitidine, led to a significant but non-sustained reduction in negative symptoms in people with schizophrenia.	Mehta and Ram, 2014
	H_3_ receptors are upregulated in the prefrontal cortex of people with schizophrenia.	Jin et al., 2009a
	H_3_ receptor antagonists improved symptoms of cognitive impairment in animal studies but this has so far not been translated to human studies.	Pre-clinical studies: Southam et al., 2009; Bardgett et al., 2010; Brown et al., 2013. Clinical trials: Haig et al., 2014; Jarskog et al., 2015

*D_2_, dopamine 2; D_3_, dopamine 3; H_1_, histamine 1; H_2_, histamine 2; H_3_, histamine 3; H_4_, histamine 4; *Hdc* KO, histamine decarboxylase knock out; HRH1, histamine receptor H1; HRH2, histamine receptor H2; HRH3, histamine receptor H3; HNMT, histamine *N*-methyltransferase.*

### Tourette’s Syndrome

Tic disorders include transient and chronic tic disorders, as well as TS that is characterized by both vocal and motor tics (Robertson, 2000). This group of neurodevelopmental disorders is typically diagnosed in childhood, with a childhood prevalence of approximately 0.6% of the population (Cleaton and Kirby, 2018). TS has numerous pre- and perinatal environmental risk factors, including parental age and education, socioeconomic status, maternal hypertension, antenatal and perinatal complications, maternal smoking and alcohol exposure and severe psychosocial stress (Chao et al., 2014). This is alongside a heritability of approximately 58% (Davis et al., 2013). Together these highlight the importance of embryogenesis and prenatal neurodevelopment in its pathophysiology. Tic disorders are commonly seen along with other comorbid mental disorders such as obsessive-compulsive disorder (OCD) and ADHD (Freeman et al., 2000). The motor and vocal tics and increased repetitive behaviors seen in TS are thought to arise from aberrant neural activity in the cortico-basal ganglia circuitry (Albin and Mink, 2006), specifically in the striatum (Jeffries et al., 2002). Histamine receptors are markedly expressed in striatum, suggesting histamine has an important role in modulation of this circuit (Bolam and Ellender, 2016; Haas and Panula, 2016; Han et al., 2020).

The hypothesis that histamine deficiency is implicated in the pathophysiology of TS stems from a seminal study in the New England Journal of Medicine by Ercan-Sencicek et al. (2010) at Yale University. This study identified a rare nonsense mutation in the gene encoding *Hdc*, the rate-limiting enzyme in histamine synthesis, as being associated with TS in a two-generation pedigree (Ercan-Sencicek et al., 2010). Subsequent genetic studies provided further evidence that dysregulated histaminergic signaling has a causal role in TS in humans through copy number variations and over-transmission of single nucleotide polymorphisms (Fernandez et al., 2012; Karagiannidis et al., 2013). The implication of the *Hdc* gene as a rare mutation has led to a monogenic causal model of TS in the form of the *Hdc* knock out (KO) mouse that is both validated and is translatable between humans and mice (Baldan et al., 2014). There is a gene copy-number dependent decrease in histamine concentration in the hypothalamus, striatum and cortex, in mice that were heterozygous or knock out for *Hdc* compared to wild type mice (Baldan et al., 2014). They did not exhibit tic-like hyperkinetic movements at rest. However, repetitive movements such as stereotypies (repetitive focused sniffing and orofacial movements) or excessive grooming, were elicited in response to acute stress and/or psychostimulant challenge that were mitigated with pre-treatment with the dopamine 2 (D_2_) receptor antagonist, haloperidol (Baldan et al., 2014). This was compared to increased locomotion in wild type mice. Both dopamine levels and D_2_/D_3_ receptor expression are increased in *Hdc* KO mice, suggesting that there may be an interaction between histaminergic and dopaminergic signaling in mediating the symptoms of TS (Baldan et al., 2014). Indeed, H_3_ receptors are found to be upregulated in *Hdc* KO mice, and chemogenetic activation of these receptors in the dorsal striatum can precipitate stereotypies (Rapanelli et al., 2017) suggesting that histamine, and specifically the H_3_ receptor in the dorsal striatum, are contributing to the repetitive movements that are a prominent feature of TS. Histamine infusion may reduce the concentration of dopamine in the striatum by agonizing H_3_ heteroreceptors on dopaminergic afferents thereby reducing onward dopaminergic signaling (Schlicker et al., 1993). However, variants in the *Hdc* gene do appear to be rare and they have not been implicated in other cases of TS in those of Caucasian and Asian origin (Lei et al., 2012) suggesting other causes. For example, Abelson et al. (2005) identified rare mutations in the Slit and Trk-like family member 1 (SLITRK1) gene in 174 unrelated TS probands/subjects. Sanger sequencing has since confirmed that rare variants in these genes are implicated in the susceptibility to TS in both a European and Canadian cohort (Alexander et al., 2016). This suggests that both can have functional roles in the pathophysiology of TS, which warrants further investigation and furthermore highlights the genetic complexity underpinning the etiology of TS.

### Autism Spectrum Disorder

Autism spectrum disorder (ASD) is a common NDD, with an estimated prevalence of 1–2% in the general population (Baird et al., 2006; Brugha et al., 2011) and a heritability of 50–60% (Buehler, 2011; Yoo, 2015). It is often diagnosed alongside multiple comorbid diagnoses of mental illness including intellectual disability (Matson and Shoemaker, 2009), TS (Kalyva et al., 2016), and schizophrenia (De Giorgi et al., 2019). ASD is characterized by impairments in social interactions and communication skills and stereotypic and restricted, repetitive behaviors and interests. It is also commonly associated with literal thinking, a lack of central coherence and difficulties with emotional regulation. The term ASD encompasses a range of presentations that in previous classification systems have been described as classical autism, pervasive developmental disorder, Asperger syndrome and atypical autism. ASD shares genetic risk factors with TS (Clarke et al., 2012; Fernandez et al., 2012) and there appears to be a marked overlap in appearance and underlying pathophysiology between stereotyped, repetitive behaviors in ASD, tics in TS and compulsions in OCD.

The etiology of ASD remains unclear. However, abnormalities in cortico-striatal circuitry have been implicated (Prat et al., 2016), as has dysregulation of several neurotransmitter systems including serotonin, acetylcholine, dopamine, GABA and glutamate (Eissa et al., 2018a). Histamine dysregulation may have a role in mediating these abnormalities with increased expression of the gene set *HNMT*, *HRH1, HRH2*, and *HRH3* seen in post-mortem brains of patients with ASD (Wright et al., 2017). In addition, the H_3_ receptor has been implicated in repetitive behavior-like pathology (Rapanelli et al., 2017). Indeed, histamine can act as a modulator of release of the aforementioned implicated neurotransmitters (Molina-Hernández et al., 2012). As mentioned above histamine can also impact the differentiation of embryonic NSCs into glutamatergic FOXP2-positive neurons. FOXP2 is a transcription factor, the expression of which influences the formation of deep cortical layers in the developing brain (Gaspard et al., 2008) with mutations resulting in abnormalities in speech and language development (Lai et al., 2001; Takahashi et al., 2003), a common feature of NDDs such ASD.

### Attention Deficit Hyperactivity Disorder

Attention deficit hyperactivity disorder (ADHD) is an NDD characterized by inattentive, hyperactive and impulsive symptoms (Luo et al., 2019). Dysregulation of dopaminergic and noradrenergic transmission has been implicated in its pathophysiology. Pharmacological treatment with stimulant and non-stimulant medications results in an increase in transmission of these catecholamines in regions of the brain associated with cognition and attention (Wilens, 2006). Histamine can also increase alertness and attention, with blockade of the H_3_ autoreceptor shown to increase histamine release and improve cognitive function (Leurs et al., 2005). The stimulant drug, methylphenidate, and non-stimulant noradrenaline reuptake inhibitor, atomoxetine, have been shown to stimulate cortical histamine release without any affinity for the H_3_ receptor (Horner et al., 2007). This may indicate that the increased release of histamine is secondary to enhanced dopamine and noradrenaline transmission. Polymorphisms of the *HNMT* gene have been seen in those with ADHD (Stevenson et al., 2010). Together, this data suggest that altered histamine transmission may be implicated in the pathophysiology of ADHD.

### Schizophrenia

Schizophrenia is severe mental illness with a lifetime prevalence of 0.7% (Saha et al., 2005). It is a chronic psychotic disorder characterized by positive symptoms such as hallucinations and delusions, negative symptoms such as social withdrawal, anhedonia and avolition, and cognition impairments. It is associated with high morbidity and mortality. While antipsychotic drugs remain the mainstay of treatment, a substantial proportion of people do not have an adequate clinical response to such medications (Howes et al., 2012a, b), necessitating a better understanding of the underlying neurobiology and the development of new approaches to treatment. The most influential theories on the neurobiology of schizophrenia are that of the dopamine and glutamate hypotheses (Howes et al., 2015). Excess dopamine transmission in the mesolimbic pathway has been associated with the positive symptoms, specifically an increase in the capacity for dopamine synthesis and release (Howes et al., 2013) and greater D_2_ receptor density (Howes et al., 2012a). However, the direction of causality is yet to be established. In addition, a substantial proportion of patients do not have a therapeutic response to dopamine modulating pharmacotherapies (Mortimer et al., 2010). The glutamate hypothesis of schizophrenia is based on the association of the disorder with NDMA receptor hypofunction and the overlap in clinical presentation with NMDA encephalitis (Howes et al., 2015). Nonetheless the precise mechanism by which altered glutamate transmission may lead to the symptoms of schizophrenia remains unclear nor are there any glutamate receptor modulating pharmacotherapies established for clinical use.

Abnormal histamine transmission has also been associated with schizophrenia. Indeed, elevated levels of the histamine metabolite tele-methylhistamine suggest greater histamine release and turnover (Prell et al., 1995, 1996). H_1_ receptor expression in cholinergic neurons in the basal forebrain is lower in patients with schizophrenia and deletion of H_1_ receptors in these neurons in mice results in a sensorimotor gating deficit, social impairment and anhedonia-like behavior (Cheng et al., 2021). H_2_ receptor antagonism has been shown to have beneficial effects in schizophrenia, including a reduction in both positive and negative symptoms (Meskanen et al., 2013; Mehta and Ram, 2014). Moreover, H_3_ receptors are upregulated in the prefrontal cortex of people with schizophrenia (Jin et al., 2009a), with initial animal studies demonstrating that H_3_ receptor antagonists improved symptoms of cognitive impairment (Southam et al., 2009; Bardgett et al., 2010; Brown et al., 2013). However, this has so far not been translated to humans with clinical trials showing no improvement in cognitive symptoms with various H_3_ receptor antagonists (Haig et al., 2014; Jarskog et al., 2015).

Together these findings would suggest that histamine is key in the normal physiological processes of the brain and is dysregulated in a variety of NDDs likely resulting in aberrant activity and/or development of key neuronal circuits. However, exactly how histamine dysregulation may alter neuronal circuitry and whether it is through a direct effect on neurons and/or indirectly through other cells often remains unclear. We will next explore how histamine is able to interact with some of the non-neuronal cells found in the brain.

## Histamine and Non-Neuronal Cells

**FIGURE 2:**
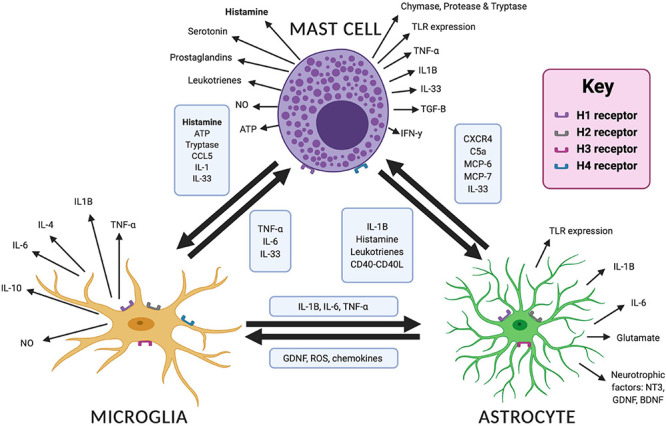
Interactions and mediators between mast cells, microglia and astrocytes in the brain. Bidirectional interactions are demonstrated between each cell type with the mediators involved in boxes. Different mediators are released in response to cellular activation, which are shown for each cell type. Image created with BioRender.com. ATP, adenosine triphosphate; BDNF, brain derived neurotrophic factor; CCL5, C–C motif chemokine ligand 5; C5a, complement 5a; CXCR4, CXC chemokine receptor 4; GDNF, glial derived neurotrophic factor; IL, interleukin; MCP, mast cell protease; NO, nitric oxide; NT3, neurotensin 3; ROS, reactive oxygen species; TLR, toll like receptor; TNF-α, tumor necrosis factor alpha.

Histamine can act as a local (paracrine) neurotransmitter as well as a modulator of the immune system. Several studies have shown that histamine can mediate a pro- or anti-inflammatory response depending on the cytokines produced and the effect of the local environment. These include pro-inflammatory cytokines such as interleukin 1-beta (IL-1β) and tumor necrosis factor alpha (TNF-α) and anti-inflammatory cytokines such as transforming growth factor beta (TGF-β) (Johnson and Krenger, 1992; Skaper et al., 2001; Dong et al., 2014a; Zhang et al., 2020). The effect of the immune system on neurodevelopment is therefore complex, promoting healthy neurodevelopment in some circumstances while being pathological in others. To complicate matters further, several cytokines, most notably interleukin 6 (IL-6), have pleiotropic functions on neuronal function (Xing et al., 1998; Scheller et al., 2011) whereby altering their precise signaling pathway may have varied effects on neurodevelopment that are yet to be fully understood. We have discussed the roles for histamine in the early development of key neural circuits. It may be that the altered development of these circuits in NDDs is compounded through neuroinflammation that can be modulated by histamine in the brain. This complex action likely involves multiple cell types (see Figure 2), including astrocytes, microglia; the resident macrophages of the brain, and mast cells; immune cells that are an important non-neuronal source of histamine. The role of these cells in neuroinflammation and neurodevelopment and the possible influence of histamine on these processes will now be discussed (see also Table 1).

### Prenatal and Postnatal Neuroinflammation and Neurodevelopment

Neurodevelopment is a highly organized process, beginning with neurogenesis and migration of young neurons followed by synaptogenesis, synaptic pruning and myelination (Knuesel et al., 2014) (see Figure 1). Every step in the formation of neuronal circuits can be disrupted by inflammation of the developing brain (Knuesel et al., 2014). Indeed, several NDDs have been associated with pre- and/or early postnatal neuroinflammation, including ASD, schizophrenia, cerebral palsy and epilepsy (Rodriguez and Kern, 2011; Knuesel et al., 2014; Kuban et al., 2015; Kim et al., 2017; Allswede and Cannon, 2018; Reed et al., 2020).

Prenatal inflammation may be mediated though increased maternal immune activation (Ji-Xu and Vincent, 2020) that has been associated with adverse neurodevelopment, particularly in the late first and early second trimester – a time at which the fetal immune system is still poorly developed (Knuesel et al., 2014). This activation of the maternal immune system may be in response to a diverse range of infections (e.g., pneumonia, sinusitis, tonsillitis, and toxoplasmosis), which may confer an increased risk of neuropsychiatric disorders (Patterson, 2009; Knuesel et al., 2014). For example, a recent study investigated the potential mechanisms underpinning this, finding that maternal immune activation can lead to an interleukin 17a (IL-17a)-dependent stress response that reduced global mRNA translation. Interestingly, this occurred in male rodents only and blockade of this stress response ameliorated any behavioral abnormalities associated with maternal immune activation, suggesting a sex-specific effect of maternal immune activation on neuroinflammation and neurodevelopment (Kalish et al., 2021). Indeed, autoimmune disorders (Patel et al., 2020) can lead to a similar sex-specific immune response. Furthermore, maternal neuronal autoantibodies have been associated with NDDs such as ASD (Dalton et al., 2003; Giannoccaro et al., 2019). For example, the contactin-associated protein-2 (CASPR2) has been identified as a specific target for maternal autoantibodies that in turn, can alter synaptic development *in utero* with lifelong behavioral abnormalities, including impaired social interactions and increased repetitive behaviors (Coutinho et al., 2017a,b,c). There are therefore multiple potential precipitants that activate the maternal immune response and confer an increased risk of NDDs.

Postnatal inflammatory illnesses are also associated with perinatal brain pathology. Indeed, precipitants such as necrotizing enterocolitis and bacteremia in neonates have been identified as risk factors for white matter damage and adverse neurodevelopmental outcomes at 2 years old (O’shea et al., 2013). However, postnatal inflammation itself may also stem from a prenatal antecedent. The Extremely Low Gestational Age Newborn (ELGAN) Study considered the risk of several prenatal antecedents of inflammation and subsequent neurodevelopment, including fetal growth restriction, maternal obesity, placental microorganisms and socioeconomic adversity. Interestingly, it found that indicators of socioeconomic disadvantage associated with an increased risk of systemic postnatal inflammation, suggesting that prenatal inflammation may persist into the postnatal period. This was also supported by the study of Yanni et al. (2017) who found that the risk of adverse neurodevelopment is heightened further when evidence of pre- and post-natal inflammation are seen together. Specifically, placental inflammation followed by persistent elevation of pro-inflammatory cytokine such as IL-6 and TNF-α was associated with a heightened risk of cerebral palsy and microcephaly at 2 years old in preterm newborns (Yanni et al., 2017).

While both antenatal and postnatal inflammation alone has been associated with adverse neurodevelopment, the maternal immune response alone has not been shown to directly cause NDDs (Bennet et al., 2018). It may be that exposure to more than one risk factor is needed for phenotypic expression, with maternal inflammation priming the individual for other genetic or environmental triggers. This hypothesis has been directly tested in animal studies where induction of maternal inflammation with lipopolysaccharide results in a hypofunction of the NMDA-receptor and loss of hippocampal synaptic plasticity. When this is combined with an environmental stressor, e.g., restraint, this led to the phenotypic expression of various ASD-related behaviors (Reisinger et al., 2015). Neuroinflammation may therefore increase the vulnerability of the brain to a second insult in the pathophysiology of NDDs.

### What Are Microglia?

Microglial cells are the antigen presenting cells of the CNS and are key mediators of neuroinflammation (Kettenmann et al., 2011). They have a role in surveillance and phagocytosis of cellular debris (Sierra et al., 2013) and maintenance of brain function and are thought to regulate many processes including neurogenesis, synaptic plasticity and synaptic pruning (Aloisi, 2001; Tremblay et al., 2010; Ji et al., 2013; Schafer and Stevens, 2013; Zhan et al., 2014; Wu et al., 2015; Bar and Barak, 2019). They are derived from primitive myeloid progenitor cells and migrate into the CNS during embryogenesis, appearing before E8 in mice and 4.5–5 weeks in humans (Lichanska and Hume, 2000; Ginhoux et al., 2010), and increase in numbers rapidly from E16 onward in mice (Swinnen et al., 2013) and are functionally heterogeneous (Smolders et al., 2019; Mendes and Majewska, 2021). Microglial progenitors infiltrate the CNS before the vasculature is maturely formed, either migrating though the ventricular walls or through the meninges (Ginhoux et al., 2010; Swinnen et al., 2013; Reemst et al., 2016). Migration and distribution may also be further regulated by direct neuronal-microglial interactions though the chemokine CX3CL1, otherwise known as Fractalkine, and its corresponding receptor expressed on the microglia (Paolicelli et al., 2011). They then disperse in non-uniform manner, comprising 0.5–16.6% of the cell population depending on the region of the adult brain (Lawson et al., 1992) and differentiate to help regulate neurodevelopment, monitoring and maintaining synapses in the healthy, uninjured brain (Aloisi, 2001; Bar and Barak, 2019). We would direct readers to some excellent review articles on the role of microglia on neurodevelopment (Cowan and Petri, 2018; Coomey et al., 2020; Thion and Garel, 2020).

### Microglia and Neuroinflammation

Increased microglial-mediated neuroinflammation has been seen in numerous NDDs, including ASD (Morgan et al., 2010; Tetreault et al., 2012; Suzuki et al., 2013; Gupta et al., 2014; Lee et al., 2017), schizophrenia (Garey, 2010; Sellgren et al., 2019; Chini et al., 2020), ADHD (Anand et al., 2017), and TS (Lennington et al., 2016). Microglia in the developing brain are also sensitive to external perturbations such as maternal infections (Smolders et al., 2015; Bernstein et al., 2016; Rosin et al., 2021a; Rosin et al., 2021b). Microglia are thought to initiate an immune response to protect the brain, but altered microglial activity has also been implicated in disorders of neurodevelopment and neurodegeneration through an upregulation of neuroinflammation (Kettenmann et al., 2011; Salter and Stevens, 2017). The precise mechanisms how a microglial bias toward pro- or anti-inflammatory cytokine production can affect neurodevelopment and pathological states remains incompletely understood. As further outlined below histamine has been shown to trigger both anti-inflammatory and pro-inflammatory responses from the microglia (Biber et al., 2007; Zhang et al., 2020). An appreciation of the role of the microglia in neuroinflammation and neurodevelopment and their regulation by histamine is therefore an exciting prospect into understanding the pathophysiology of a range of neurodevelopmental disorders.

### Microglia and NDDs

**FIGURE 3:**
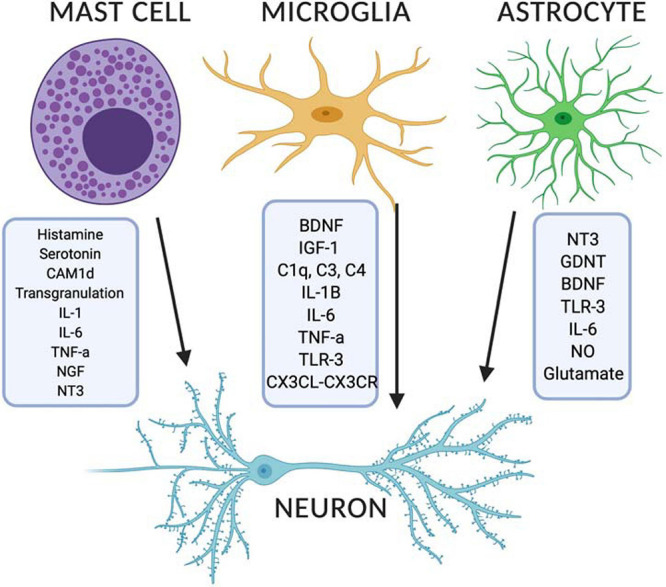
Released modulators from mast cells, microglia and astrocytes can impact on developing neurons in the brain. Image created with BioRender.com. BDNF, brain derived neurotrophic factor; CX3CL, CX3C chemokine ligand 1; CX3CL, CX3C chemokine receptor; GDNF, glial derived neurotrophic factor; IGF-1, insulin-like growth factor 1; IL, interleukin; NGF, nerve growth factor; NO, nitric oxide; NT3, neurotensin 3; TLR, toll like receptor; TNF-α, tumor necrosis factor alpha.

Microglia have been implicated in neurodevelopmental disorders such as ASD and schizophrenia, both of which are characterized as having deficits in synaptic pruning, synaptogenesis and altered circuit development. Proposed mechanisms include those mediated through the action of complement proteins such as C1q, C3, and C4 (Stevens et al., 2007; Paolicelli et al., 2011; Fu et al., 2012; Ramaglia et al., 2012; Schafer et al., 2012) and/or altered release of brain derived neurotrophic factor (BDNF) with deficits in AMPA and NMDA receptor-mediated long-term potentiation (LTP) and synaptic remodeling (Parkhurst et al., 2013) (see Figure 3). Moreover, *HoxB8* knock out mice, a gene expressed by a subpopulation of microglia, has been associated with increased grooming behaviors similar in nature to TS, OCD and ASD (Chen et al., 2010) and as seen in the *Hdc* KO model of TS. These behaviors are then attenuated by repopulation of the brain with wild type microglia (Chen et al., 2010). Lastly, there is emerging evidence on the importance of CD4+ T cells in microglial activation in facilitating normal neurodevelopment (Pasciuto et al., 2020). Although not discussed here in detail T cells are interesting in that they are able to control brain inflammatory responses through histaminergic signaling (Jutel et al., 2001; del Rio et al., 2012; Korn and Kallies, 2017) and could facilitate cross-talk between the brain and the periphery (Rustenhoven et al., 2021) in conjunction with other factors (Salvador et al., 2021).

### Histamine’s Regulation of Microglia

Microglia have been shown to express all four subsets of histamine receptor (Dong et al., 2014a; Haas and Panula, 2016; Zhang et al., 2020), which can differentially affect their behavior. For example,Frick et al. (2016) undertook one of the first *in vivo* studies on the role of histamine in microglial activation. The group used immunohistochemistry to assess the effect of either histamine deficiency (*Hdc* KO mouse model) or histamine stimulation in wild type mice on microglia. Histamine was shown to regulate microglia via the H_4_ receptor. *Hdc* KO mice have a normal number of microglia but with reduced ramifications, reduced insulin-like growth factor-1 (IGF-1) expression and reduced expression of H_4_ receptor that may indicate impairment in histaminergic regulation of microglia. Similar findings occurred by selective removal of histaminergic neurons in the TMN of the hypothalamus. IGF-1 expressing microglia are induced by cytokines released from T helper 2 cells, which may be neuroprotective, promoting neurogenesis. Furthermore, the pro-inflammatory microglial response to challenge with lipopolysaccharide (LPS) was greater in *Hdc* KO mice. This may indicate that a genetic predisposition such as histamine deficiency may increase the brain’s vulnerability to pro-inflammatory insults in neuropsychiatric disorders such as TS. Indeed exogenous histamine was able to reduce LPS induced inflammation in the hippocampus (Saraiva et al., 2019).

Interestingly, *in vitro* studies have shown both pro- and anti-inflammatory effects of histamine on microglial function (Biber et al., 2007). For example, histamine can reduce pro-inflammatory cytokine production such as IL-1β in response to mediators such as LPS as well modulate overall microglial motility (Ferreira et al., 2012). This may indicate an anti-inflammatory function of histamine on the microglia. However, in contrast to this, microglial secretion of the pro-inflammatory cytokines TNF-α and IL-6 is triggered by histaminergic stimulation of the H_1_ and H_4_ receptors (Dong et al., 2014a; Zhu et al., 2014). Specifically, Zhang et al. (2020) found that histamine could induce microglial activation and the production of the pro-inflammatory cytokines TNF-α and IL-1β that was partially negated with H_1_ and H_4_ receptor antagonists and stimulated with H_1_ and H_4_ receptor agonists. On the other hand, H_2_ and H_3_ receptor antagonists led to significant increases in TNF-α and IL-1β, and H_2_ and H_3_ receptor agonists significantly increased the release of the anti-inflammatory cytokine interleukin-10 (IL-10). This is further supported by both Chen et al. (2020) who found that H_2_ and H_3_ receptor agonism inhibited laparotomy- or LPS-induced microglial activation, pro-inflammatory cytokine production and cognitive decline and by Fang et al. (2020) whereby H_4_ receptor antagonism reduced microglial activation and TNF-α release in a rat model of Parkinson’s disease. Along with histamine’s ability to induce microglial activation and the subsequent release of both anti- and pro-inflammatory factors, it can also promote phagocytosis via H_1_ receptor activation and the production of reactive oxygen species (Rocha et al., 2016) and prostaglandin E2 (Lenz et al., 2018).

Overall, these findings highlight the pleiotropic nature of the microglia in mediating their immune response and suggest potential roles for histamine in regulating microglial-mediated inflammation. What remains poorly studied though, is which sources of histamine within the CNS may contribute to both microglia-mediated neuroinflammation and altered neurodevelopment.

### What Are Astrocytes?

Astrocytes are the most numerous cell type found within the CNS, forming complex networks with neuronal and non-neuronal cells alike. They are dynamic cells that have a wide range of functions and are fundamental for brain homeostasis (Nedergaard et al., 2003; Escartin et al., 2021). During early neurodevelopment, astrocytes have a trophic effect, facilitating the generation and migration of neuronal cells, facilitating synaptogenesis and the creation and maintenance of neuronal circuits (Ricci et al., 2009). One astrocyte can communicate with multiple neurons, with most of these structures being tripartite in nature; structural units formed of pre- and postsynaptic components of two neurons and an astrocyte (Araque et al., 1999; Halassa et al., 2007; Cavaccini et al., 2020). They can sense and respond to changes in the local microenvironment (e.g., local neurotransmitters) to control neuronal signaling (Wahis et al., 2021) and protect neurons from oxidative damage and neuronal injury. They are also important in energy metabolism (Brown and Ransom, 2007; Parra-Abarca et al., 2019), ionic homeostasis (Olsen et al., 2015), blood flow regulation (Howarth, 2014) and the formation of the blood brain barrier and can release gliotransmitters such as adenosine triphosphate (ATP), glutamate and D-serine (Parpura et al., 1994; Zhang et al., 2003; Volterra and Meldolesi, 2005; Hamilton and Attwell, 2010).

### Astrocytes and Neuroinflammation

There is a growing evidence to suggest that astrocytes can modulate the immune system within the CNS and are important in regulating neuroinflammation (Rothhammer and Quintana, 2015). We have already discussed that neuroinflammatory processes can have both protective or detrimental effects on the developing brain. So too can astrocytic activation (Cekanaviciute and Buckwalter, 2016). Astrocyte activation can lead to the release of trophic factors such neurotrophin-3 (NT-3), glial cell line-derived neurotrophic factor (GDNF) and BDNF (Jurič et al., 2011; Thomsen et al., 2017). These growth-promoting molecules promote neuronal survival and GDNF has also been shown to inhibit microglial activation (Ossola et al., 2011; Rocha et al., 2012) resulting in a dampening down of neuroinflammation. Conversely, astrocytic activation can also lead to pro-inflammatory cytokine release, alongside increased concentrations of chemokines and reactive oxygen species and microglial activation (Sochocka et al., 2017) resulting in increased excitotoxicity, apoptosis and neurodegeneration. The mechanisms underlying the induction of a specific neuroinflammatory process remains poorly understood. Recent cutting-edge approaches have started to describe key target molecules important for the interactions between astrocytes and microglia in these neuroinflammatory processes (Clark et al., 2021). It is hoped that improved understanding of astrocyte-microglia cross-talk may then reveal new potential therapeutic targets for modulation that could be relevant in an array of neurodevelopmental disorders.

### Histamine’s Regulation of Astrocytes

The H_1_, H_2_, and H_3_ receptors are expressed on astrocytes (Jurič et al., 2016) (see Figure 2), though H_3_ receptor expression may be restricted to certain brain regions and may vary depending on the species that is studied (Karpati et al., 2018). Our current understanding of how astrocytes can respond to histaminergic activity in the brain originated over three decades ago (Hosli and Hosli, 1984; Hosli et al., 1984) and continue to be investigated. For example, Karpati et al. (2018) employed the human astrocytoma cell line 1321N1 to better establish the underlying mechanism and found that histamine can interact with astrocytic histamine receptors resulting in glutamate release in an H_1_ receptor-dependent and concentration-dependent manner suggesting that histamine can form part of neuron-astrocyte communications.

There is less data available if and how histaminergic activity might influence astrocytic immunomodulation. Some studies have shown that histamine can act synergistically with pro-inflammatory cytokines such as IL-1 (Lipnik-Stangelj and Carman-Krzan, 2006) and IL-6 (Lipnik-Štangelj and Čarman-Kržan, 2005; Ales et al., 2008) to modulate astrocytic release of neurotrophins such as NGF. For example, Xu et al. (2018) investigated the role of histamine on astrocytic neuromodulation and neuroprotection. They found that histamine selectively upregulated the expression of H_1_, H_2_, and H_3_ receptors, stimulated the synthesis of astrocytic GDNF and inhibited the production of pro-inflammatory cytokines, TNF-α and IL-1β in a concentration-dependent manner. The increased production of neurotrophic factors likely highlights an important mechanistic role in CNS recovery from injury by promoting neuronal survival and synaptogenesis (Lipnik-Stangelj and Carman-Krzan, 2004; Jurič et al., 2011; Xu et al., 2018). We have already discussed that released GDNF can inhibit microglial activation *in vivo* and *in vitro*, thereby revealing a possible interaction between these glial cells in modifying (microglial-mediated) neuroinflammation (Rocha et al., 2012; Zhang et al., 2014). In addition see also recent findings suggestive of purinergic signaling from astrocytes to microglia upon histaminergic stimulation (Xia et al., 2021). Moreover, changes in astrocyte-neuronal crosstalk have been implicated in the development of mental disorders, including depression, ASD and schizophrenia (Roman et al., 2020). However, to our knowledge, there are no studies that have investigated the specific role of histamine in directly modulating astrocytic behavior contributing to neurodevelopmental disorders.

### Mast Cells as a Non-neuronal Source of Histamine

Mast cells are immune cells derived from hematopoietic precursors, originating within the bone marrow from CD34+/CD117+ pluripotent progenitors (Gilfillan et al., 2011). They then mature within the microenvironment of various tissues, including the vascular endothelium and the brain, where they participate in both innate and adaptive immune responses, even in the absence of antigen presentation (Dong et al., 2014b). Mast cells in general express H_1_ and H_4_ receptors, which have been implicated in the pathophysiology of peripheral type 1 hypersensitivity reactions and increased histamine and cytokine generation, respectively (Thangam et al., 2018) and guide chemotaxis (Hofstra et al., 2003; Halova et al., 2012). However, their expression in brain mast cells has yet to be confirmed. Mast cells are located in perivascular regions within close vicinity of neurons, especially in the hypothalamus, the pineal and pituitary glands (Theoharides, 2017), velum interpositum below the hippocampus (Panula et al., 2014), the meninges (Reuter et al., 2001; Galli et al., 2005a) and are able to cross the normal blood brain barrier (Silverman et al., 2000). The ability to traverse the blood brain barrier may be accentuated further by disease states affecting its integrity which can intimately be linked to mast cell activation and contribute to neuroinflammation and neurotoxicity (Theoharides et al., 2012), including during periods of neurodevelopment. Approximate mast cell numbers in the developing rodent brain have recently been described and are mainly localized to the pia mater and the thalamus. Within the pia mater, mast cells are most numerous during early development, with approximately 3,500 seen at birth, peaking at approximately 5,000 at postnatal day 11. Numbers then decline to approximately 1,500 at P15, though the remaining mast cells become more concentrated in the pia that overlies the anterior thalamus. The total numbers of mast cell within the pia then reach adult levels of approximately 50 by P30. Within the thalamus, around 140 mast cells are seen at P8, which then steadily increases to reach adult values of 1,500 at P30 (Khalil et al., 2007; Panula et al., 2014).

Mast cells produce a range of mediators, some of which are preformed, whereas others are synthesized upon activation. These mediators include the biogenic amines histamine and serotonin, cytokines, specifically IL-1, IL-6, TNF-α, interferon-γ (IFN-γ), TGF-β, enzymes such as phospholipases, chymase, and mast cell proteases and tryptase, lipid mediators such as leukotrienes and prostaglandins, growth factors, nitric oxide, heparin, ATP and neuropeptides (Johnson and Krenger, 1992; Skaper et al., 2001; Dong et al., 2014b). Despite their small numbers they can affect numerous processes in the brain that have a potentially underestimated impact on neuroinflammation (see Figure 2). Preformed mediators may be released from secretory granules within seconds, followed by *de novo* formation of lipid mediators, cytokines and chemokines (Galli et al., 2005b; Nelissen et al., 2013; Silver and Curley, 2013). Mast cells are a heterogeneous cell type, with wide variation in mediator synthesis and release and a wide response in signaling pathways (Dong et al., 2014b) some of which seems to depend on histamine synthesis by mast cells itself (Ohtsu et al., 2001). Mast cells are an important source of histamine in the brain, with up to 50% of brain histamine levels in rodents attributable to the presence of mast cells (Yamatodani et al., 1982). This was established using high-performance liquid chromatography in mast cell deficient (Kit^*W/Wv*^) mice compared to controls at 2–4 months after birth (Yamatodani et al., 1982). Such mice have reduced *c-kit* tyrosine kinase-dependent signaling, leading to impaired mast cell development and survival (Kitamura et al., 1978; Grimbaldeston et al., 2005). These mice are profoundly deficient in mast cells, with adult mice containing no detectable mast cells across numerous anatomical sites by 6–8 weeks of age (Kitamura et al., 1978).

### Mast Cell Interactions With Microglia

Mast cells are a non-neuronal source of histamine that can be released upon degranulation. We have already discussed the role of histamine-mediated microglial activation and the release of the pro-inflammatory cytokines IL-6 and TNF-α *in vitro* via H_1_ and H_4_ receptors and MAPK and PI3K/AKT pathway activation (Dong et al., 2014a). Other implicated pathways include the complement 5a receptor and chemokine receptor 4/12 (CXCr4 and CXCL12) (Dong et al., 2014a) and the chemoattractant, C-C Motif Chemokine Ligand 5 (CCL5) (Hendriksen et al., 2017). We can therefore see an array of *in vitro* evidence for bidirectional interactions between mast cells and microglia in regulating neuroinflammation some of which are highlighted below (see also Figure 2).Dong et al. (2017) provided the first data on *in vivo* mast cell-microglial interactions, demonstrating that activation of brain mast cells by injecting the mast cell degranulator, C48/80 directly into the hypothalamus triggered microglial activation and the release of the pro-inflammatory cytokines, IL-6 and TNF-α. In turn, this was opposed by mast cell stabilization using sodium cromoglycate. Indeed, this resulted in a decrease in pro-inflammatory cytokines and reduced expression of the innate immune protein, toll-like receptor 4 (TLR4), and H_1_ and H_4_ receptors on the microglia. In turn, there was no effect on microglial activation in mast-cell deficient Kit^*W–sh/W/–sh*^ mice. Similar to the Kit^*W/Wv*^ mice discussed previously, these mice have reduced *c-kit* tyrosine kinase-dependent signaling, leading to impaired mast cell development and survival (Kitamura et al., 1978; Grimbaldeston et al., 2005). However, the specific mutation used is thought to lead to fewer developmental abnormalities that the Kit^*W/Wv*^ model while still retaining the desired mast cell deficiency (Yamazaki et al., 1994; Grimbaldeston et al., 2005). The findings by Dong et al. (2017) are important not only in confirming an interaction between mast cells and microglia *in vivo*, but also in highlighting the importance of mast cell degranulation for this interaction. Given that mast cell activation may be the first responder to injury (Jin et al., 2009b), and not the microglia, inhibition of mast cell activation may inhibit the pro-inflammatory cascade and therefore protect against neuroinflammation. What remains poorly understood is the contribution and role of histamine, if any, in this interaction. However, as the altered microglial expression of the H_1_ and H_4_ receptors depends on the activation state of mast cells (Dong et al., 2017) this may be suggestive that mast cell sources of histamine, not just neuronal sources, are crucial in the initiation of neuroinflammation.

### Mast Cell Interactions With Astrocytes and Neurons

As well as mast cell-microglial interactions, there is some emerging evidence that mast cells may have direct interactions with CNS neurons and astrocytes as outlined below. Indeed, mast cells tend to co-localize with neurons (Skaper et al., 2012; Silver and Curley, 2013) or even to strongly adhere to neurons (Hagiyama et al., 2011). Neuronal release of neuropeptides such as NGF, neurotensin and substance P have been shown to bind directly bind to mast cells, altering their activation state (Kulka et al., 2008). Conversely, mast cells may also communicate with neurons via transgranulation, whereby mast cell granules can be inserted into adjacent neurons that alters neuronal responsiveness to its microenvironment (Wilhelm et al., 2005) (see Figure 3). Kempuraj et al. (2019) investigated such interactions in a mouse model of Parkinson’s disease. They found that mouse mast cell protease-6 and 7 induced the release of interleukin 33 (IL-33) from astrocytes and a mixed culture of glia and neuronal cells. This suggested that mast cells might interact with astrocytes and neurons to accelerate neuroinflammation and neurodegeneration. Kim et al. (2010) investigated the signaling pathways of activated mast cells and their interaction with astrocytes in experimental allergic encephalomyelitis. This was used as a model for the chronic demyelinating disease, multiple sclerosis. Co-culturing of mast cells with astrocytes led to increased release of histamine, leukotrienes and pro-inflammatory cytokines. It does so via enhanced expression of CD40L on mast cells, which is the natural ligand for CD40 expressed on astrocytes. This CD40-CD40L may therefore be important in chronic disease associated with neuroinflammation. Lenz et al. (2018) investigated the role of mast cells, and specifically histamine released from mast cell degranulation on neuronal development in the preoptic area of the hypothalamus. This is a crucial brain region in determining sexual behavior. Mast cell activation with the estrogen steroid hormone, estradiol, was found to stimulate microglial activation, subsequent prostaglandin release which was associated with increased dendritic spine density and the dendritic spine protein, spinophilin, as well as more masculinized sexual behavior. A small number of mast cells therefore had a profound effect on overall brain development and resultant behavior. To our knowledge, there are no further studies that have investigated the effect of mast cell activation and non-neuronal histamine directly on the CNS *in vivo*. However, bi-directional communication was recently demonstrated between mast cells and neurons in the skin (Zhang et al., 2021), which may demonstrate a role in the mediation of epidermal and dermal inflammation. Mast cell sources of histamine have also been implicated in the pathophysiology of neuropathic pain (Rosa and Fantozzi, 2013).

### Mast Cells and Neurodevelopmental Disorders

Given their numerous cellular interactions and their role in neuroinflammation, it has been postulated that brain mast cells may be implicated in NDDs such as ASD (Theoharides et al., 2013) and ADHD (Song et al., 2020) but further roles in the etiology of NDDs remain unclear. The association with ASD is suggested by findings of mast cell related markers such as elevated serum neurotensin (Carraway et al., 1982; Alysandratos et al., 2012; Tsilioni et al., 2014), proinflammatory cytokines (Li et al., 2009) and the chemoattractant, monocyte chemoattractant protein-1 (MCP-1) (Vargas et al., 2005) in brain parenchyma and cerebrospinal fluid in patients with ASD. Secondly, ADHD has been commonly seen alongside allergic, inflammatory and autoimmune diseases (Song et al., 2020) and children with atopic eczema may be more susceptible to developing ADHD (Genuneit et al., 2014). This, along with the previously discussed associations between ADHD and neuroinflammation, has led to interest in mast cells as an important mediator of this (Song et al., 2020). However, to our knowledge, there are no studies that have investigated the potential role, if any, of mast cells in ADHD specifically.

## Histamine as a Therapeutic Target in Neurodevelopmental Disorders

So far, we have discussed that histamine is not only a neuromodulator but can be a modulator of neuroinflammation and can be part of the complex interaction between mast cells, microglia and astrocytes. Interactions between these various cells are well suited to modulate many aspects of brain development. As such histamine may be a potential therapeutic target for pharmacological manipulation to either prevent or treat the signs and symptoms of a variety of neurodevelopmental disorders (NDDs). Here we will discuss a range of compounds that have been developed to act at histamine receptors and are being investigated in the management of NDDs (see Table 3). Although often assumed to be acting mainly at neurons it remains possible that non-neuronal cells are also involved.

**TABLE 3:** Summary of preclinical and clinical studies of histamine receptor modulators in neuropsychiatric disease.

**Drug**	**Mechanism of action**	**Study design**	**Finding**	**References**
Hydroxyzine	H_1_ receptor antagonist	Case report: male patient with a rare, mis-sense mutation in *HNMT* associated with a severe intellectual disability.	Hydroxyzine and a low histidine diet reduced aggression, improving speech development and sleep disturbance.	Verhoeven et al., 2020
Famotidine	H_2_ receptor antagonist	Randomized, double-blind, placebo-controlled, cross-over design of 9 children with ASD.	Four of 9 children randomized had evidence of behavioral improvement. Children with marked stereotypy did not respond.	Linday et al., 2001
Famotidine	H_2_ receptor antagonist	Double-blind, placebo-controlled, parallel-group, randomized trial of famotidine in treatment-resistant schizophrenia.	Famotidine did not lead to a significant improvement in Scale for the Assessment of Negative Symptoms score. However, the Positive and Negative Syndrome scale total score and the Clinical Global Impression scale showed significantly greater change in the famotidine group than in the placebo group. No significant adverse effects were observed.	Meskanen et al., 2013
Famotidine	H_2_ receptor antagonist	Three-week, open-label study of famotidine (20 mg twice a day) was added as an adjunctive medication in people with schizophrenia and schizoaffective disorder.	Total Brief Psychiatric Rating Scale and Clinical Global Impression scores were significantly lower during the 3 weeks with famotidine compared with the week before and after its administration. Negative symptoms as measured by the Schedule for the Assessment of Negative Symptoms were not significantly different during famotidine treatment.	Deutsch et al., 1993
ABT-239 and A-431404	H_3_ receptor antagonists	Preclinical study using rats administered ketamine or MK-801 to induce cognitive impairments as a model of schizophrenia.	Chronic, but not acute, treatment with ABT-239 significantly improved spontaneous alternation impairments, suggesting that H_3_ receptor antagonists may have the potential to ameliorate cognitive deficits in schizophrenia.	Brown et al., 2013
ABT-288	H_3_ receptor antagonist	Preclinical study: *in vitro* and *in vivo* pharmacological profile of ABT-288 in rats (P20–24)	ABT-288 improved social recognition, spatial learning and reference memory with good pharmacokinetics and oral bioavailability of 37–66%. There was a wide central nervous system and cardiovascular safety margin.	Esbenshade et al., 2012
ABT-288	H_3_ receptor antagonist	A multicenter, randomized, double-blind, placebo-controlled, parallel-group 12-week study of ABT-288 (10 or 25 mg) vs. placebo in clinically stable subjects with schizophrenia (*n* = 214).	Study medication was tolerated. There was an increased incidence of psychosis-related and sleep-related adverse events associated with ABT-288. Neither dose of ABT-288 resulted in cognitive improvement in clinically stable adults with schizophrenia.	Haig et al., 2014
ABT-288	H_3_ receptor antagonist	Randomized, double-blind, placebo- and active-controlled (donepezil) phase 2 study of ABT-288 in subjects with mild-to-moderate Alzheimer’s disease (*n* = 242)	ABT-288 did not have pro-cognitive efficacy in subjects with mild-to-moderate Alzheimer’s Disease, but was safe and well tolerated.	Haig et al., 2012
ABT-288	H_3_ receptor antagonist	Randomized, double-blind, placebo-controlled, dose-escalating study designs of the safety and tolerability ABT-288 in young adults and in elderly subjects.	Single doses up to 40 mg and doses up to 3 mg once-daily taken over 12 (for elderly subjects) or 14 days (for younger subjects) were generally safe and well tolerated. Based on the above results, 1 and 3 mg once-daily doses of ABT-288 were advanced to phase 2 evaluation in Alzheimer’s patients.	Othman et al., 2013
ABT-288	H_3_ receptor antagonist	Randomized, double-blind, placebo-controlled, dose-escalating study of ABT-288 (10 dose levels, from 1 to 60 mg once daily for 14 days) in stable subjects with schizophrenia treated with an atypical antipsychotic (*n* = 67).	ABT-288 was tolerated at a 15-fold higher dose and 12-fold higher exposures in subjects with schizophrenia than previously observed in healthy volunteers. ABT-288 was generally safe and tolerated at doses up to 45 mg once daily.	Othman et al., 2014
Bavisant (JNJ-31001074)	H_3_ receptor antagonist	Randomized, double-blind, placebo- and active-controlled, parallel-group, multicenter study evaluated three dosages of bavisant (1, 3, or 10 mg/day) and two active controls in adults with ADHD.	Bavisant, a highly selective, wakefulness-promoting H_3_ antagonist, did not display significant clinical effectiveness in the treatment of adults with ADHD.	Weisler et al., 2012
Ciproxifan	H_3_ receptor antagonist	Preclinical study of mice with ASD-like behaviors induced by prenatal exposure to valproic acid (VPA).	VPA animals presented a significantly higher nociceptive threshold. Ciproxifan was not able to modify this parameter but was able to attenuate sociability deficits and stereotypies present in the VPA model of autism.	Baronio et al., 2015
Ciproxifan	H_3_ receptor antagonist	Preclinical study using MK-801 to mimic the hypoglutamatergic state suspected to exist in schizophrenia.	H_3_ antagonists can alleviate the impact of NMDA receptor hypofunction on some forms of memory, but may exacerbate its effect on other behaviors.	Bardgett et al., 2010
DL77	H_3_ receptor antagonist	Preclinical study of mice with ASD-like behaviors induced by prenatal exposure to valproic acid (VPA).	DL77 improved sociability and social novelty preference and attenuated the release of proinflammatory cytokines following lipopolysaccharide challenge.	Eissa et al., 2018b
GSK207040	H_3_ receptor antagonist	Preclinical study using rats with deficits in novel object recognition memory and pre-pulse inhibition induced by isolation rearing, and hyperlocomotor activity induced by amphetamine.	GSK207040 significantly enhanced object recognition memory and attenuated isolation rearing-induced deficits in pre-pulse inhibition but did not reverse amphetamine-induced increases in locomotor activity.	Southam et al., 2009
GSK239512	H_3_ receptor antagonist	Phase II randomized controlled trial of GSK239512 vs. placebo in cognitive impairment in 50 stable outpatients with schizophrenia.	GSK239512 was generally well tolerated with an adverse event profile consistent with the known class pharmacology of H_3_ receptor antagonists. There was no evidence of overall beneficial effects of GSK239512 for cognitive impairment in this population.	Jarskog et al., 2015
GSK239512	H_3_ receptor antagonist	Part A was a single-blind, placebo run-in, flexible dose titration over 9 days in two cohorts, each consisting of two patients. Part B was a double-blind, randomized, placebo controlled, parallel group, which investigated 3 flexible dose titration regimens over 4 weeks in 3 cohorts, each consisting of eight patients.	GSK239512 displayed a satisfactory level of tolerability in patients with Alzheimer’s disease with evidence for positive effects on attention and memory.	Nathan et al., 2013
JNJ-10181457	H_3_ receptor antagonist	Preclinical study to evaluate the behavioral and neurochemical effects of JNJ-10181457 in rats.	Selective blockade of H_3_ receptor might have therapeutic utility for the treatment of working memory deficits and learning disorders, especially those associated with reduced cholinergic neurotransmission.	Galici et al., 2009
SAR110894	H_3_ receptor antagonist	Preclinical study evaluating the ability of SAR110894 to inhibit tau pathology and prevent cognitive deficits in a tau transgenic mouse model (THY-Tau22)	SAR110894 treatment for 6 months decreased tau hyperphosphorylation in the hippocampus and the formation of neurofibrillary tangles in the cortex, hippocampus, and amygdala. SAR110894 also prevented episodic memory deficits, and this effect was still detected after treatment washout.	Delay-Goyet et al., 2016
Thioperamide	H_3_ receptor antagonist	Preclinical study investigating the effect of thioperamide on memory consolidation and recall mechanisms in rats.	H_3_ receptor antagonism improves memory retention and reverses the cognitive deficit induced by scopolamine in a two-trial place recognition task	Orsetti et al., 2001
Thioperamide and ciproxifan	H_3_ receptor antagonists	Preclinical study using mice with natural deficits in pre-pulse inhibition as a model of schizophrenia.	Thioperamide and ciproxifan both improved natural deficits in pre-pulse inhibition in mice suggesting that they may have therapeutic potential in the treatment of schizophrenia.	Browman et al., 2004
E100	Dual-active H_3_ receptor antagonist and AChE inhibitor	Preclinical study of mice with ASD-like behaviors induced by prenatal exposure to valproic acid (VPA).	E100 dose-dependently ameliorated repetitive and compulsive behaviors. Pretreatment with E100 attenuated anxiety levels, microglial activation, proinflammatory cytokine release and expression of NF-κB, iNOS, and COX-2 in the cerebellum.	Eissa et al., 2019
E100	Dual-active H_3_ receptor antagonist and AChE inhibitor	Preclinical study of mice with ASD-like behaviors induced by prenatal exposure to valproic acid (VPA).	E100 dose-dependently attenuated sociability deficits and mitigated oxidative stress status by increasing the levels of decreased glutathione, superoxide dismutase, and catalase in VPA mice.	Eissa et al., 2020a
E100	Dual-active H_3_ receptor antagonist and AChE inhibitor	Preclinical study of BTBR mice, a confirmed model of autism	E100 dose-dependently attenuated social deficits of BTBR mice, repetitive/compulsive behaviors and reduced the number of activated microglial cells compared to the saline-treated BTBR mice. Numbers of activated microglial cells were entirely reversed by co-administration of an H_3_ receptor agonist.	Eissa et al., 2020b
JNJ7777120	H_4_ receptor antagonist	Preclinical study of BTBR mice, a confirmed model of autism	Decreased expression of the pro-inflammatory cytokines IL-17 and IL-22 and increased Foxp3-producting CD8+ T cells, suggesting that H_4_ receptor antagonism may block inflammatory signaling in the brain.	Ahmad et al., 2019

*AChE, acetylcholinesterase; ASD, autism spectrum disorder; BTBR, *BTBR* T^+^Itpr3^*tf*^/J *mouse*; COX-2, cyclo-oxygenase-2; HNMT, histamine *N*-methyltransferase; H_1_, histamine 1; H_2_, histamine 2; H_3_, histamine 3; H_4_, histamine 4; IL-17, interleukin-17; IL-22, interleukin-22; iNOS, inducible nitric oxide synthase; NF-κB, nuclear factor-κB; VPA, valproic acid.*

### H_1_ Receptor Antagonists

The only study so far to use an H_1_ receptor antagonist in the treatment of a NDD is a case report by Verhoeven et al. (2020). They describe a male with a rare missense mutation in the *HNMT* gene located in chromosome 2q22.1 that resulted in reduced histamine metabolism within the CNS. This was associated with a severe intellectual disability. The combination of a low histidine diet and the H_1_ receptor antagonist, hydroxyzine, led to an improvement in sleep, speech development and reduction in aggression. While H_1_ receptor antagonists such as promethazine may also be used in the symptomatic treatment of insomnia, there is no evidence of such agents having a disease modifying effect in NDDs.

### H_2_ Receptor Antagonists

The H_2_ receptor antagonist, famotidine, has been trialed for treatment of social deficits that can be a clinical feature of both ASD and schizophrenia. The rationale was based on a case report by Kaminsky et al. (1990) who described incidental improvements in social deficits in an adult with schizophrenia when treated with famotidine for peptic ulcer disease. Subsequent studies then observed benefits when used as an adjunct treatment in schizophrenia with doses up to 100 mg per day (Deutsch et al., 1993; Oyewumi et al., 1994; Rosse et al., 1995; Linday et al., 2001). However, this improvement in social function has not been consistently replicated in randomized trials. Famotidine did not benefit those with ASD and marked stereotypies (Linday et al., 2001). The authors did not provide an explanation for this, but did discuss the difficulties with subtyping disorders such as ASDs based on behavior alone. In addition, while scores on the Clinical Global Impression (Deutsch et al., 1993; Meskanen et al., 2013), Total Brief Psychiatric Rating (Deutsch et al., 1993) and Positive and Negative Syndrome (Meskanen et al., 2013) scales improved in those with schizophrenia treated with adjunct famotidine, no improvements were seen in scales for negative symptoms (Deutsch et al., 1993; Meskanen et al., 2013). Given the H_2_ receptor inverse agonism noted with the antipsychotic, clozapine (Humbert-Claude et al., 2012), the authors speculated that famotidine’s H_2_ receptor antagonism might, at least in part, have an antipsychotic effect (Meskanen et al., 2013). It may also be that famotidine improves sociability in people with schizophrenia by alleviating anxiety associated with positive symptoms rather than a disease modulating effect on negative symptoms. However, the potential mechanism(s) for this have not been established.

### H_3_ Receptor Antagonists

The H_3_ receptor antagonists/inverse agonists are the most widely studied group of agents. Pitolisant is the only such agent licensed for use in adults and approved for the treatment of excessive daytime sleepiness for those with narcolepsy with or without cataplexy. It has high affinity and selectivity for the H_3_ receptor. Specifically, the administration of a dose of 40 mg of pitolisant has been shown to lead to H_3_ receptor occupancy of 84 ± 7% at the time of peak plasma concentration (Rusjan et al., 2020). Pitolisant induces central histaminergic and noradrenergic transmission in animal models that increase wakefulness and decrease REM sleep. Several studies have demonstrated its safety and tolerability (Lin et al., 2008; Inocente et al., 2012; Harwell and Fasinu, 2020) before the HARMONY I (Dauvilliers et al., 2013), HARMONY III (Dauvilliers et al., 2019), and Harmony-CTP trials (Szakacs et al., 2017) demonstrated its efficacy. It has also been shown to reduce excessive daytime sleepiness in obstructive sleep apnea (Dauvilliers et al., 2020), though is not clinically recommended as an alternative to existing treatments that target the underlying cause and multisystem consequences of obstructive sleep apnea such as continuous positive airway pressure (Trotti, 2020). Moreover, Pitolisant has shown procognitive efficacy in children with Prader Willi syndrome (PWS). Approximately 65% of those with PWS will experience sleep disturbance such as sleep apnea or daytime sleepiness (Pullen et al., 2019). It was this that prompted interest into the potential role of pitolisant given its approval in treating narcolepsy with or without cataplexy. However, for those with PWS, the benefits experienced appeared to extend beyond that of alleviating excessive daytime sleepiness (Pullen et al., 2019). From this, there is now increasing interest in epigenetic approaches that combine H_3_ receptor antagonism/inverse agonism with inhibitors of histone H3 lysine-9 (H3K9) methyltransferase G9a with the aim of restoring expression of the candidate PWS genes on the maternally inherited chromosome (Reiner et al., 2020). It is hoped that such approaches may help improve the profiles of further H_3_ receptor antagonist/inverse agonists where thus far, there has been limited reproducibility of findings from preclinical to clinical studies. While animal models have shown a potential role for pitolisant in other conditions such as epilepsy (Sadek et al., 2014), this has so far not translated to benefit in human studies (Kasteleijn-Nolst Trenite et al., 2013).

Altered sleep-wake cycles are common in those with ASD, including circadian rhythm disorders, dyssomnias and parasomnias (Souders et al., 2017; Hohn et al., 2019; Ballester et al., 2020; Jovevska et al., 2020). To our knowledge, there have not been studies into the use of pitolisant as a wake promoting agent specifically in those with NDDs. However, other H_3_ receptor antagonists/inverse agonists are in development for other indications relevant to those with NDDs. Most these have focused on the modulating restricted and repetitive behaviors or aggression or at improving cognition and sociability in animal models (Orsetti et al., 2001; Galici et al., 2009; Southam et al., 2009; Esbenshade et al., 2012; Brown et al., 2013; Baronio et al., 2015; Eissa et al., 2018a, b, 2020b). For example, a clinical trial with the H_3_ receptor antagonist, AZD5213, for people with TS showed a 2–3 point reduction in Total Tic Severity Score Compared to placebo (Clinical Trials.Gov, 2016). However, one study that investigated the use of three doses of the H_3_ receptor antagonist, bavisant, in adults with ADHD did not find any clinical benefit (Weisler et al., 2012). The lower doses of 1 and 3 mg/day were well tolerated, but 10 mg/day was associated with higher rates of discontinuation due to adverse effects (particularly sleep-related adverse effects). In comparison, methylphenidate 54 mg/day and atomoxetine 80 m/day were more efficacious than placebo. The authors concluded that direct modulation of dopamine and noradrenaline transmission may be better than histamine modulation in the alleviation of ADHD symptoms. Furthermore, preclinical studies demonstrated procognitive effects of the H_3_ receptor antagonist, ABT-288 (Esbenshade et al., 2012). A phase 1 study of adjunct treatment using ABT-288 was tolerated at a dose 15 times higher in humans with schizophrenia than had previously been seen in healthy volunteers (Othman et al., 2014). However, a 12-week phase 2, multicenter, randomized controlled trial of ABT-288 (10 mg, 25 mg or placebo) did not demonstrate a pro-cognitive effect in people with schizophrenia (Haig et al., 2014). Overall, while these agents appear well tolerated in human studies (Dauvilliers et al., 2013; Othman et al., 2013, 2014; Szakacs et al., 2017; Dauvilliers et al., 2019, 2020), there is limited consistent data to suggest they are efficacious in treating symptoms seen in NDDs (Haig et al., 2012, 2014; Weisler et al., 2012; Jarskog et al., 2015). Indeed, the contrast between positive preclinical findings with negative clinical trials may also reflect the challenges in finding translatable animal models for neuropsychiatric disorders (Howe et al., 2018).

### H_4_ Receptor Antagonists

The expression of the H_4_ receptor on microglia and mast cells led to interest in the use of H_4_ receptor antagonists in reducing neuroinflammation in the treatment of NDDs such as ASD. For example, Ahmad et al. (2019) used one such experimental H_4_ receptor antagonist, which led to decreased expression of pro-inflammatory cytokines. However, this study did not ascertain if this effect was associated with an amelioration of symptoms associated with ASD. The selective H_3_ receptor antagonist, clobenpropit, has also shown off-target H_4_ receptor partial agonism (Patnaik et al., 2018). Like the H_3_ receptor inverse agonist, BF 2649, this agent led to a reduction in beta amyloid deposits and gliosis in a rat model of Alzheimer’s disease (Patnaik et al., 2018). It is unclear if and to what extent the H_4_ receptor affinity may contribute to this potential neuroprotective effect. To our knowledge, there are no H_4_ receptor modulators that have been tested in clinical trials.

It is important to note that those with NDDs tend to be under-represented in clinical trials. The reasons for this may be due to concerns over the high frequency of comorbidity that may limit the internal validity of any findings, concerns over capacity to consent, particularly those with a comorbid intellectual disability, and the resources to adapt written information and data collection tools to promote participation in those with are non-verbal. While existing trials are focusing on repurposed agents such as the diuretic bumetanide, the vasopressin 1a receptor agonist, balovaptan and the GABA_*B*_ receptor agonist, baclofen, modulation of histamine receptors may be an additional viable target in helping to ameliorate difficulties associated with NDDs.

## Conclusion

In this Review, we have highlighted the importance of the biogenic amine, histamine, in modulating the development of key neuronal circuits that are implicated in neurodevelopmental disorders, such as ASD and TS amongst others, as well as regulating inflammatory processes in the brain. Most studies have focused on neuronal sources of histamine but what remains unclear is to what extent other sources of histamine, for example from mast cells, participate in these processes and allow for interactions between neuronal and non-neuronal cells. While existing studies have been predominantly *in vitro*, there is some, albeit limited, *in vivo* data that indicates that mast cell degranulation and the release of preformed mediators, such as histamine, can influence microglial-mediated neuroinflammation in a bidirectional manner. There is also evidence of direct interactions between mast cells and astrocytes and neurons, which may be through the release of non-neuronal histamine. However, to our knowledge, there are no studies that investigate the specific role of mast cell sources of histamine on modulating both neuroinflammation and its subsequent impact on synaptic development in key neural circuits that underpin NDDs *in vivo*. This therefore represents an important area of future research as early modulation of mast cell function may provide a novel therapeutic target for NDDs including TS, ASD, ADHD and schizophrenia. In addition, the potential therapeutic role of histamine receptor modulators and the well-tolerated group of H_3_ receptor antagonists/inverse agonists may represent a promising area in the management of NDDs.

## Author Contributions

EC wrote the first draft of the manuscript. EC and TE wrote sections of the manuscript. Both authors contributed to manuscript revision, read, and approved the submitted version.

## Conflict of Interest

The authors declare that the research was conducted in the absence of any commercial or financial relationships that could be construed as a potential conflict of interest.
